# Ferroptosis reshapes the tumor immune microenvironment: molecular mechanisms, immune regulation, and therapeutic synergistic strategies

**DOI:** 10.3389/fimmu.2026.1772259

**Published:** 2026-02-27

**Authors:** Zhuangwei Lv, Zixian Liu, Ziru Nie, Ning Li, Lingbei Kong, Fangfang Shen, Junna Jiao, Hui Wang

**Affiliations:** 1 School of Forensic Medicine, Xinxiang Medical University, Xinxiang, China; 2 School of Basic Medical Sciences, Xinxiang Medical University, Xinxiang, China; 3 The Key Laboratory for Tumor Translational Medicine, The Third Affiliated Hospital of Xinxiang Medical University, Xinxiang, Henan, China; 4 Xinxiang Engineering Technology Research Center of Immune Checkpoint Drug for Liver-Intestinal Tumors, Xinxiang Medical University, Xinxiang, China; 5 Henan Key Laboratory of Immunology and Targeted Drugs, Xinxiang Medical University, Xinxiang, Henan, China; 6 Department of Immunology, Henan Collaborative Innovation Center of Molecular Diagnosis and Laboratory Medicine, School of Medical Technology, Xinxiang Medical University, Xinxiang, Henan, China

**Keywords:** cancer therapy, ferroptosis, *GPX4*, lipid peroxidation, YAP signaling pathways

## Abstract

This article systematically reviews ferroptosis—an iron-dependent, lipid peroxidation-driven form of programmed cell death. It provides a detailed analysis of its core regulatory mechanisms, encompassing the drive from lipid peroxidation, the collapse of antioxidant defenses such as the glutathione peroxidase 4(*GPX4*)axis and alternative pathways like Ferroptosis Suppressor Protein 1 (*FSP1*), and the remodeling of iron and lipid metabolism. The interplay between ferroptosis and other cell death modalities, such as apoptosis and necroptosis, is also elucidated. The review focuses on the pivotal roles of key signaling pathways, including *NRF2, p53, and Hippo-YAP*, within the ferroptosis regulatory network. In the context of cancer therapy, the article emphasizes the potential of inducing ferroptosis for reversing drug resistance, inhibiting metastasis, and synergizing with immunotherapy. It systematically outlines direct induction strategies (e.g., small-molecule inducers, nanodelivery systems) and combination strategies with conventional therapies, targeted therapy, and immunotherapy. This review highlights that the bidirectional interplay between ferroptosis and the tumor immune microenvironment constitutes a novel therapeutic paradigm for combination therapy. Specifically, it elucidates how ferroptosis modulates immune cells such as CD8^+^ T cells and macrophages, reshaping the tumor immune microenvironment and offering new avenues for combination immunotherapy. We conclude by providing a roadmap for translating these insights into clinical practice, addressing current challenges, and outlining future directions for developing next-generation anticancer strategies.

## 1 Introduction

Ferroptosis is an iron-dependent form of regulated cell death driven by lipid peroxidation, distinct from apoptosis, necrosis, and autophagy in its morphological, biochemical, and regulatory mechanisms. The concept was first proposed in 2012 by Brent Stockwell’s team, based on the unique cell death phenotype induced by the small-molecule compounds Erastin and RSL3 (1). Initially identified in screens targeting RAS-mutant cancer cells, these compounds were found to trigger a non-apoptotic, iron-dependent cell death through distinct mechanisms, which was subsequently defined as ferroptosis (1).

Hallmarks of ferroptosis include glutathione depletion, inactivation of glutathione peroxidase 4 (*GPX4*), accumulation of reactive oxygen species (ROS), and peroxidation of polyunsaturated fatty acids(PUFAs) (2–4). Beyond cancer biology, ferroptosis is implicated in diverse physiological (e.g., development, immune regulation) and pathological processes (e.g., neurodegenerative diseases, ischemia-reperfusion injury) (5–7). Particularly in oncology, ferroptosis represents a promising avenue for overcoming therapy resistance by modulating tumorigenesis, metastasis, and treatment response. For example, cancer cells with RAS or TP53 mutations, metastatic cells, and therapy-persistent cells often demonstrate increased susceptibility to ferroptosis (4).

The induction of ferroptosis shows significant potential for clinical translation in cancer therapy. Resistance to conventional chemotherapy or radiotherapy frequently involves upregulation of antioxidant pathways (e.g., the NRF2 and System Xc^−^-GSH-GPX4 axis), rendering such cells highly vulnerable to ferroptosis (8, 9). Furthermore, combining ferroptosis inducers (e.g., sorafenib, artesunate derivatives) with immune checkpoint inhibitors(*ICIs*) can synergistically activate anti-tumor immune responses by promoting the release of tumor antigens and damage-associated molecular patterns (10). Nanodelivery systems (e.g., PLGA nanoparticles) enable targeted delivery of ferroptosis inducers, enhancing therapeutic efficacy while reducing systemic toxicity (8).

This review aims to systematically analyze the molecular mechanisms of ferroptosis (lipid peroxidation drive, collapse of antioxidant defenses, and regulation of iron/lipid metabolism), its crosstalk with other cell death pathways, and key regulatory networks (e.g., *NRF2, p53, Hippo-YAP* pathways). Particular emphasis is placed on how ferroptosis remodels the tumor immune microenvironment, influencing immune cells such as CD8^+^ T cells, macrophages, and myeloid-derived suppressor cells. It will also critically evaluate recent advances in targeting ferroptosis to reverse therapy resistance, inhibit metastasis, and enhance immunotherapy (e.g., *GPX4* inhibitors, ferritinophagy activators, metabolic interventions), while addressing challenges in clinical translation (e.g., biomarker scarcity, targeting selectivity). The review seeks to provide a theoretical foundation for developing next-generation anticancer therapies (11, 12).

## 2 Core regulatory mechanisms of ferroptosis

### 2.1 Lipid peroxidation: the execution engine of ferroptosis

Lipid peroxidation serves as the direct driver of ferroptosis, centered on the oxidative damage to phospholipids containing polyunsaturated fatty acids (PUFAs). Iron ions generate reactive oxygen species via the Fenton reaction, initiating a free radical chain reaction that ultimately forms lethal lipid hydroperoxides (13, 14). Phospholipids with PUFAs, such as phosphatidylethanolamine, are particularly vulnerable due to their double-bond structures. Arachidonoyl- and adrenoyl-phosphatidylethanolamines are identified as key substrates (15–17).

PUFAs are esterified into phospholipids through a process involving acyl-CoA synthetase long-chain family member 4 (ACSL4), which links them to coenzyme A, and lysophosphatidylcholine acyltransferases (LPCATs). Consequently, ACSL4 and LPCATs are crucial determinants of cellular sensitivity to ferroptosis (18). These enzymes are essential for the execution of ferroptosis upon GPX4 inhibition, while ACSF2 is required for erastin-induced ferroptosis (1, 19).

Lipoxygenases (ALOXs) represent an enzyme family that catalyzes PUFA oxidation by introducing hydroperoxyl groups into fatty acid chains, thereby initiating lipid peroxidation. The six human ALOX isoforms, with their distinct substrate preferences and catalytic activities, contribute to ferroptosis in different cell types or tissues (20, 21). ALOX15/15B directly catalyze PUFA oxidation, whereas cytochrome P450 reductase and NADPH oxidase indirectly promote this process by generating an oxidative environment (10, 22). In contrast, MBOAT1 and MBOAT2 suppress ferroptosis by remodeling the cellular phospholipid profile to generate monounsaturated fatty acid-PE species.

Mitochondria, as a primary source of ROS, exhibit characteristic changes during ferroptosis, including increased membrane density and reduced cristae. Enhanced oxidative phosphorylation in these organelles can further accelerate lipid peroxidation (10). Notably, membrane damage triggered by ferroptosis can be mitigated through repair by the ESCRT-III complex, representing a key cellular resistance mechanism (10). The lipid peroxidation products generated during ferroptosis can act as damage-associated molecular patterns (DAMPs), which are critical for activating immune cells such as dendritic cells and macrophages, thereby linking ferroptosis execution to antitumor immunity.

### 2.2 Collapse of antioxidant defenses: the *GPX4* axis and alternative pathways

*GPX4* is the key enzyme in the antioxidant defense against ferroptosis, reducing lipid hydroperoxides to alcohols within biological membranes (23). However, three independent systems that suppress ferroptosis independently of GPX4 have been identified: FSP1/coenzyme Q10, dihydroorotate dehydrogenase (DHODH), and GCH1/BH4.

**Figure 1:**
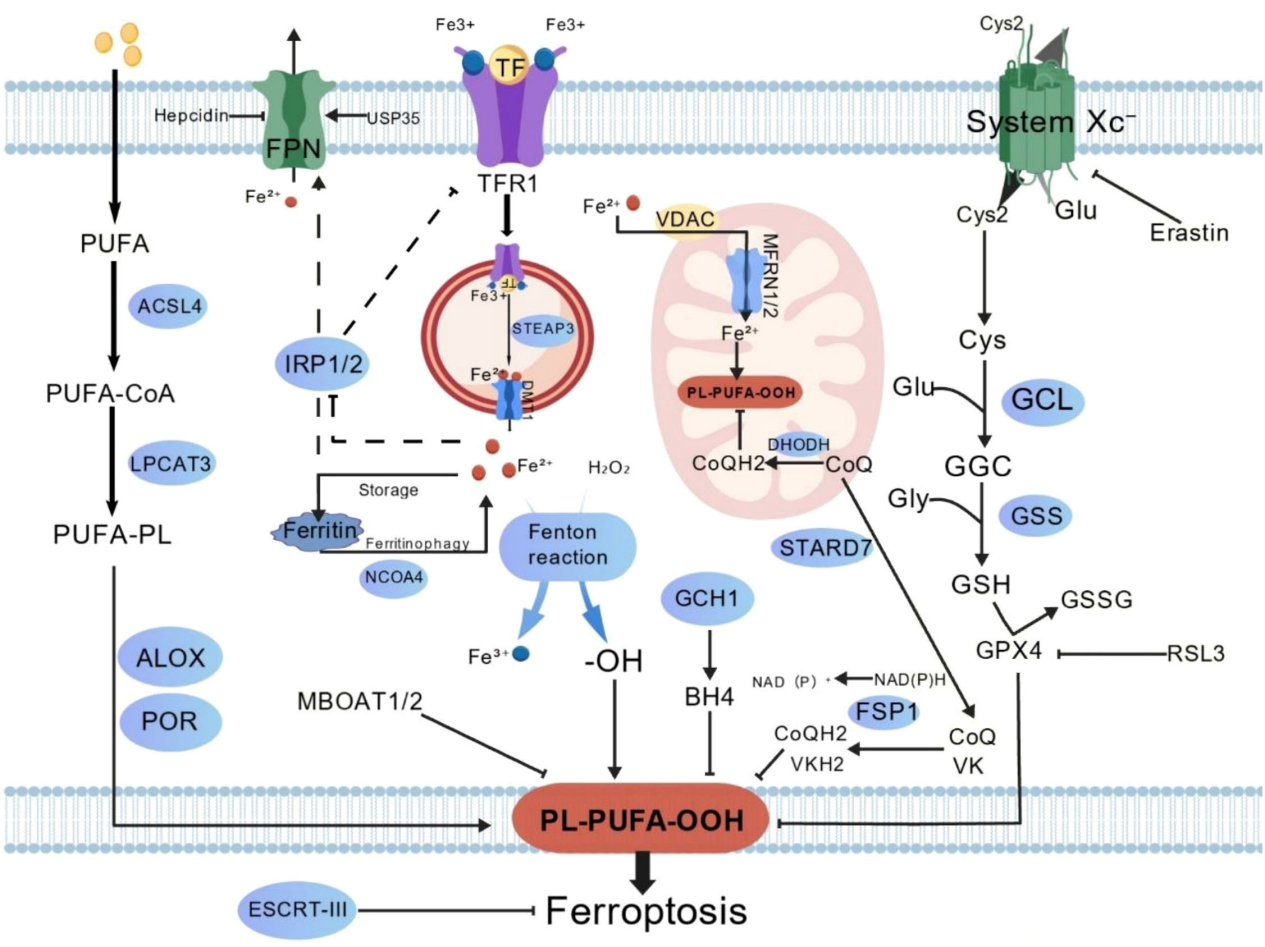
Mechanisms of ferroptosis regulation. The core regulatory network of ferroptosis involves a balance between driving forces and antioxidant defenses. The primary defense is the System Xc–GSH-GPX4 axis, which reduces toxic lipid hydroperoxides, with parallel pathways like FSP1-CoQ10 and GCH1-BH4 providing additional protection. Key drivers include intracellular iron, which catalyzes peroxidation via the Fenton reaction, and the enzymatic incorporation and oxidation of PUFAs into membrane phospholipids. The process culminates in the execution phase, where excessive lipid peroxide accumulation causes membrane damage and cell death, a fate that can be counteracted by membrane repair systems like ESCRT-III.

The System Xc–GSH-GPX4 axis constitutes the primary defense system against lipid peroxidation. System Xc–, composed of solute carrier family 7 member 11(SLC7A11) and solute carrier family 3 member 2 (SLC3A2) subunits, mediates the cystine/glutamate exchange, providing the precursor for glutathione synthesis (24). GSH serves as an essential cofactor for GPX4, enabling the reduction of lipid hydroperoxides to non-toxic alcohols (25). Disruption of this axis by GPX4 inhibitors (e.g., RSL3, ML162) or System Xc– inhibitors (e.g., Erastin, sulfasalazine) induces ferroptosis (26). Evidence demonstrates that genetic inhibition of GPX4 triggers ferroptosis in tumor cells and suppresses tumor growth *in vivo (*23). These findings underscore the significant role of the canonical System Xc–GSH-GPX4 regulatory pathway in tumor biology (**Figure 1**).

Beyond the canonical pathway, three distinct GPX4-independent systems suppressing ferroptosis have been identified. The ferroptosis suppressor protein 1(FSP1)/coenzyme Q_10_ system constitutes a primary parallel defense: localized at the plasma membrane, FSP1 functions as a CoQ oxidoreductase that utilizes NAD(P)H to reduce CoQ to ubiquinol (CoQH_2_), which acts as a lipophilic radical-trapping antioxidant to inhibit ferroptosis (27, 28). The ferroptosis-suppressing capacity of FSP1 is dependent on a functional CoQ biosynthesis pathway (27, 28).

Two additional pathways provide further defense: GTP cyclohydrolase 1 (GCH1) protects against lipid peroxidation through the synthesis of tetrahydrobiopterin, while DHODH sustains the mitochondrial CoQH_2_ antioxidant pool (28–30). DHODH, a mitochondrial enzyme involved in pyrimidine biosynthesis, influences ferroptosis susceptibility in cancer cells with low GPX4 expression, likely by utilizing coenzyme Q10 as an electron acceptor during its catalytic cycle (31). Elevated expression of either GCH1 or DHODH confers stronger resistance to ferroptosis, whereas their lower expression increases cellular sensitivity (15). The recently identified MBOAT1/2 enzymes enhance membrane antioxidant capacity by remodeling the phospholipid profile, independently of sex hormone receptor regulation (28). The discovery of these alternative pathways not only expands the defensive dimension against ferroptosis but also suggests that targeting GPX4 alone may be insufficient for complete ferroptosis induction. Future therapeutic strategies should explore concurrent targeting of multiple pathways, particularly in treatment-resistant tumors. Importantly, these antioxidant pathways are often upregulated in immunosuppressive cells (e.g., myeloid-derived suppressor cells) within the tumor microenvironment, and their inhibition can simultaneously induce ferroptosis and reverse immune evasion, offering a dual therapeutic benefit.

### 2.3 Regulation of iron metabolism–the death catalyst

As an iron-dependent form of cell death, ferroptosis is characterized by an expanded labile iron pool (LIP), primarily comprising Fe²^+^. Cellular iron uptake is largely mediated by the binding of serum transferrin to transferrin receptor 1 (*TFR1*) and subsequent endocytosis (32). Intracellular iron homeostasis is maintained through multi-layered regulation: *TFR1* facilitates external iron acquisition, while the divalent metal transporter promotes intracellular iron release and ferroportin exports iron under hepatic regulation (33, 34).

Degradation of the iron storage protein ferritin occurs via *NCOA4-*mediated ferritinophagy, releasing free iron that directly fuels the Fenton reaction. Notably, *NCOA4* overexpression alone can elevate the intracellular LIP by enhancing ferritin degradation (10, 35). The iron regulatory protein/iron-responsive element (IRP/IRE) system senses cellular iron levels and dynamically coordinates the expression of genes such as *TFR1* and *FPN (*29). Furthermore, mitochondrial iron metabolism, through its role in heme synthesis, also influences the progression of ferroptosis (12). Iron availability also modulates immune cell functions; for instance, iron overload can promote M2 macrophage polarization, whereas iron restriction may enhance T cell responses, indicating that targeting iron metabolism could reshape the tumor immune landscape.

### 2.4 Lipid metabolic remodeling–a key determinant of cellular susceptibility

The phospholipid composition of cellular membranes directly determines susceptibility to ferroptosis. Acyl-CoA synthetase long-chain family member 4 (ACSL4) catalyzes the conjugation of PUFAs to coenzyme A, forming PUFA-CoAs, which are subsequently incorporated into membrane phospholipids by lysophosphatidylcholine acyltransferase 3 (LPCAT3) (10, 16). High ACSL4 expression significantly increases ferroptosis sensitivity, whereas its deficiency confers resistance (16).

Lipid metabolism interacts with ferroptosis through multiple pathways: Stearoyl-CoA desaturase-1 (SCD1) generates monounsaturated fatty acids that substitute for PUFAs, thereby reducing lipid peroxidation substrates. Conversely, lipophagy promotes ferroptosis by degrading lipid droplets and releasing free fatty acids (22). Recent studies reveal that MBOAT1/2 inhibit ferroptosis, independent of the GPX4/FSP1 pathway, by remodeling the phospholipid profile through the reacylation of lysophospholipids (28). Furthermore, sterol O-acyltransferase 1 (SOAT1) suppresses ferroptosis by reducing free cholesterol levels, uncovering the complexity of the lipid metabolic network in regulating this cell death process (36).

### 2.5 Ferroptosis and metabolic reprogramming

#### 2.5.1 Mitochondrial metabolism and ferroptosis

Mitochondria are the central organelles for cellular energy metabolism. They are responsible for the tricarboxylic acid (TCA) cycle, oxidative phosphorylation (OXPHOS), and the generation of ROS. Consequently, mitochondrial metabolism plays a pivotal role in the regulation of ferroptosis. Studies have shown that cysteine deprivation can lead to mitochondrial membrane hyperpolarization and accumulation of lipid peroxides, thereby inducing ferroptosis (37, 38). Inhibiting the TCA cycle or OXPHOS can alleviate this process (38). Pyruvate is a key substrate for the TCA cycle. It is converted to acetyl-CoA via pyruvate dehydrogenase (PDH), thereby driving the TCA cycle. In pancreatic ductal adenocarcinoma, pyruvate dehydrogenase kinase 4 (PDK4) enhances resistance to ferroptosis by inhibiting pyruvate oxidation.This reduces acetyl-CoA production and fatty acid synthesis (39). Furthermore, under hypoxic conditions, lactate dehydrogenase (LDH) promotes the conversion of pyruvate to lactate. This process diminishes the support of glucose aerobic oxidation for ferroptosis. Lactate can also activate the sterol regulatory element-binding transcription factor 1 (SREBP1)/stearoyl-CoA desaturase 1 (SCD1) axis. This stimulates the generation of monounsaturated fatty acids (MUFAs). MUFAs replace polyunsaturated fatty acids in the cell membrane, alleviating lipid peroxidation and thereby resisting ferroptosis (40).

#### 2.5.2 Regulatory mechanisms of glucose metabolism and ferroptosis

In glucose metabolism, key glycolytic enzymes HK2 and PKM2 finely regulate ferroptosis sensitivity by modulating metabolic flux and redox homeostasis. HK2 not only drives glycolysis but also influences ROS production and mitochondrial function through its localization at mitochondria, thereby participating in the regulation of cell death processes (41, 42). PKM2 exhibits a dual role: its tetrameric form promotes glycolytic flux and may support antioxidant synthesis, whereas the dimeric form can translocate to the nucleus and modulate stress-responsive genes; studies have confirmed that PKM2 can directly regulate ferroptosis by affecting mitochondrial homeostasis (43, 44), highlighting its potential as a metabolic checkpoint in cancer therapy (45).

**Figure 2:**
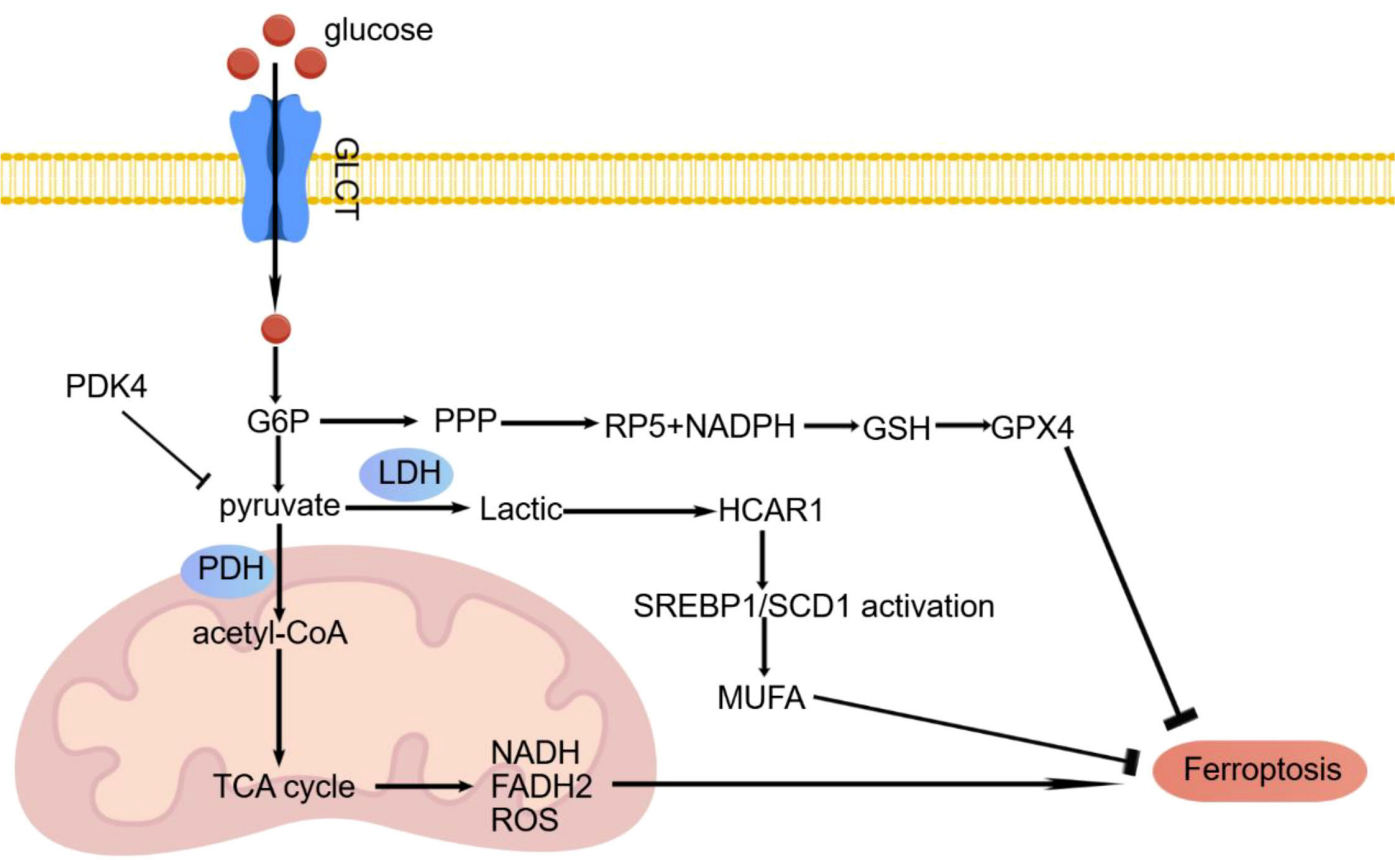
A simplified schematic diagram illustrating the regulation of ferroptosis by key glucose metabolism pathways. This diagram summarizes the core pathways within glucose metabolism that influence ferroptosis. Glucose generates NADPH via the pentose phosphate pathway, which is utilized for glutathione synthesis, thereby supporting the antioxidant function of GPX4 to inhibit ferroptosis. Pyruvate, produced through glycolysis, serves as a crucial metabolic node: it can enter the mitochondrial TCA cycle via PDH, generating ROS to promote ferroptosis; alternatively, under the regulation of PDK4 or hypoxic conditions, it can be converted to lactate by LDH. Lactate, either transported intracellularly or present in the microenvironment, can be sensed by the surface receptor HCAR1. This interaction activates the downstream SREBP1/SCD1 signaling axis, which promotesmonounsaturated fatty acid synthesis and consequently inhibits lipid peroxidation, enhancing resistance to ferroptosis.

The pentose phosphate pathway (*PPP*) is a crucial branch of glucose metabolism, generating NADPH and pentose phosphates (**Figure 2**). As an electron donor, NADPH reduces oxidized glutathione (GSSG) to reduced glutathione (GSH) under the action of glutathione reductase (GR). GSH is an essential cofactor for *GPX4*, which inhibits ferroptosis by eliminating lipid peroxides (46). In tumor cells, even under ample oxygen supply, there is a preference for generating energy via glycolysis, a phenomenon known as the Warburg effect. The *PPP* not only provides precursors for nucleotide synthesis but also produces substantial NADPH, supporting rapid tumor cell proliferation (47). Additionally, NADPH is involved in GSH synthesis, aiding tumor cells in resisting oxidative damage (48). In pancreatic cancer, upstream stimulatory factor 2 (USF2) binds to the promoter of pyruvate kinase M2 (PKM2), upregulating PKM2 expression. This subsequently increases intracellular GSH levels and GPX4 activity, thereby inhibiting ferroptosis (43). Recent studies have also indicated that mucosal melanoma cells can acquire resistance to ferroptosis by taking up lactate. This enhances the PPP pathway, upregulates GPX4 and FSP1, and increases levels of NADH, NADPH, and GSH (49).

#### 2.5.3 Regulatory mechanisms of amino acid metabolism and ferroptosis

**Figure 3:**
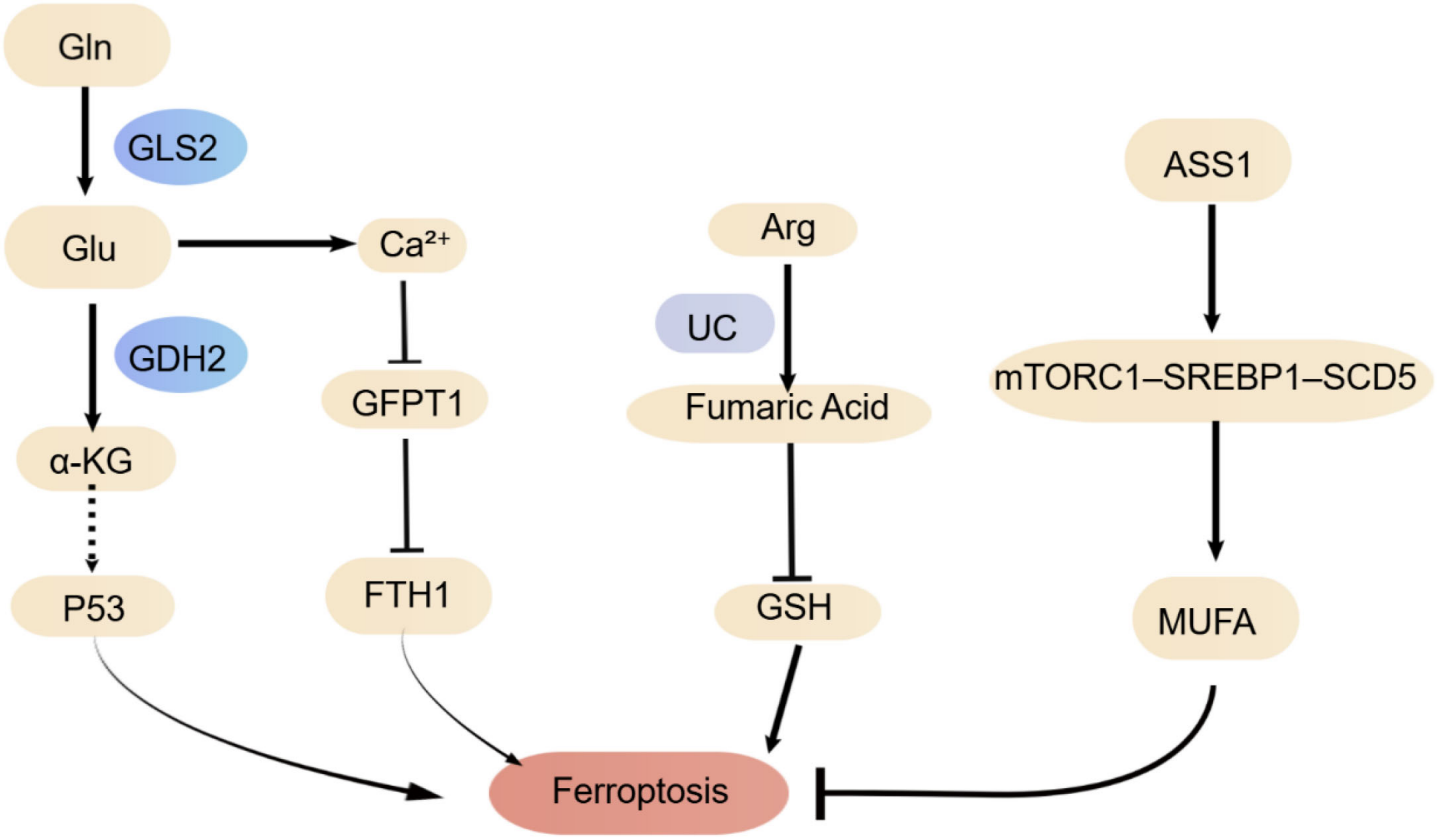
Ferroptosis and amino acid metabolic pathways.

Ferroptosis, an iron-dependent form of programmed cell death, is closely associated with various amino acid metabolic processes (**Figure 3**). Among these, glutamine metabolism plays a central role in the regulation of ferroptosis. After entering the cell via the transporter SLC1A5, glutamine is converted to glutamate by glutaminase (GLS). Glutamate can then be further catalyzed by glutamate dehydrogenase 1 (GDH1) to generate A-ketoglutarate (A-KG), thereby driving glutaminolysis. Intracellular glutamate accumulation inhibits system Xc– activity, reducing cystine uptake, which leads to impaired glutathione (GSH) synthesis and diminished antioxidant capacity. Elevated endogenous glutamate levels cause an increase in intracellular Ca^2+^ concentration, subsequently activating the adenylate cyclase (ADCY10)-protein kinase A (PKA) signaling axis. This phosphorylates and inhibits GFPT1, thereby weakening YAP protein stability and the transcription of its downstream target gene FTH1, ultimately promoting ferritinophagy, lipid peroxidation, and enhanced ferroptosis susceptibility (50). Furthermore, A-KG can be further metabolized to 2-hydroxyglutarate, inducing mitochondrial ROS accumulation and activating the p53 pathway, collectively promoting ferroptosis (51). Notably, GLS2, an isoenzyme of GLS, exhibits dual roles in liver cancer by suppressing tumors and promoting ferroptosis (52). Therefore, targeting key nodes in glutamine metabolism, such as SLC1A5, GLS, or GDH1, has become a significant strategy for regulating ferroptosis (53, 54).

Besides glutamine, arginine metabolism also plays a complex role in ferroptosis regulation. On one hand, arginine can be metabolized via the urea cycle to generate fumarate. As an electrophile, fumarate covalently binds to glutathione, leading to intracellular GSH depletion and thereby promoting erastin-induced ferroptosis (55). On the other hand, the metabolic pathway mediated by argininosuccinate synthase 1 (ASS1) can antagonize ferroptosis. ASS1 promotes the reductive carboxylation of glutamine, reducing its entry into the TCA cycle for oxidative metabolism, consequently lowering the accumulation of mitochondria-derived lipid ROS. Simultaneously, ASS1 participates in the arginine-succinate metabolic axis, activating the mTORC1–SREBP1–SCD5 signaling pathway, which promotes monounsaturated fatty acid synthesis, enhances the anti-peroxidation capacity of the cell membrane, and ultimately confers resistance to ferroptosis in tumor cells (56). Thus, the role of amino acid metabolism in regulating ferroptosis is dependent on the metabolic context and molecular mechanisms. Different metabolic enzymes and microenvironments can lead to divergent cell fates, providing a theoretical basis and potential targets for intervening in ferroptosis via specific amino acid pathways.

Glutamine is imported via SLC1A5 and metabolized to glutamate and A-ketoglutarate through GLS/GDH1. This process promotes ferroptosis through multiple mechanisms: inhibiting system Xc^−^, depleting GSH, activating p53, and suppressing the GFPT1-YAP-FTH1 signaling axis via the ADCY10/PKA pathway. In contrast, the metabolic pathway mediated by ASS1 confers resistance to ferroptosis by promoting the reductive carboxylation of glutamine and activating the mTORC1-SREBP1-SCD5 axis to drive monounsaturated fatty acid synthesis. However, arginine can also be metabolized via the urea cycle to generate fumarate, which acts as an electrophile to deplete glutathione, thereby promoting ferroptosis.

### 2.6 Crosstalk between ferroptosis and other cell death modalities

Ferroptosis does not occur in isolation but forms an intricate cell death network with other regulated cell death pathways, including apoptosis, necroptosis, pyroptosis, and the recently identified cuproptosis.

#### 2.6.1 Intersection with apoptosis

Despite distinct execution mechanisms, ferroptosis and apoptosis interact at multiple levels. They share upstream stress signals; for instance, the p53 tumor suppressor can transcriptionally repress SLC7A11 to promote ferroptosis while also initiating classical apoptosis (57). Mitochondria serve as a key hub: outer membrane permeabilization and collapse of membrane potential are hallmark apoptotic events that also cause ROS bursts, exacerbating ferroptosis (58). In myocardial ischemia/reperfusion injury, Tanshinone IIA concurrently inhibits both caspase-3-mediated apoptosis and GPX4 degradation-triggered ferroptosis by targeting VDAC1, highlighting the mitochondrial channel’s coordinating role (59).

Their relationship is dynamic and can be mutually restrictive. Activated caspases can suppress ferroptosis by cleaving glutaminase, thereby limiting glutamate availability essential for System Xc^−^ function (60). Therapeutically, co-induction can overcome resistance. In breast cancer, DNAJC12 concurrently suppresses doxorubicin-induced apoptosis and ferroptosis via the HSP70-AKT axis, while combining inhibitors for both pathways fully reverses chemoresistance (61). Similarly, in oral squamous cell carcinoma, melatonin synergizes with erastin to induce both apoptosis and ferroptosis via ROS promotion (62). Several studies indicate that the efficacy of traditional chemotherapeutics partly stems from their ability to activate both pathways (63).

#### 2.6.2 Synergy with necroptosis

Ferroptosis and necroptosis often act synergistically in acute tissue injury, amplifying inflammation and damage. Metabolic crosstalk is central: activation of the key necroptotic kinases RIPK1/RIPK3 can cause energy crisis and ROS bursts, creating conditions favorable for ferroptosis (64). Conversely, severe oxidative stress from ferroptosis can upstream activate the RIPK3-MLKL pathway (65).

In acute kidney injury, a “wave-like death” model proposes that initial ferroptosis acts as an “initiator,” releasing DAMPs that subsequently activate necroptosis in neighboring cells as an “amplifier” to propagate death signals, a process termed “necroinflammation” (66). In acute respiratory distress syndrome (67), intestinal ischemia/reperfusion injury (68), and calcium oxalate crystal-induced kidney injury (69), ferroptosis, necroptosis, and pyroptosis frequently co-occur, forming a highly interconnected, functionally redundant network. Inhibiting one pathway often leads to “compensatory” death via others, presenting a therapeutic challenge and pointing to the need for multi-pathway targeting.

#### 2.6.3 Link to pyroptosis

Ferroptosis and pyroptosis are closely linked through inflammation. Lipid peroxides accumulated during ferroptosis act as DAMPs, activating the NLRP3 inflammasome (70). Activated caspases-1/4/5/11 then cleave gasdermin D (GSDMD); its N-terminal fragment forms pores in the plasma membrane, executing pyroptosis and releasing pro-inflammatory cytokines like IL-1β, thereby coupling oxidative damage to intense inflammation (71).

In epilepsy models, ferroptosis and pyroptosis jointly contribute to disease progression via oxidative stress and neuroinflammation, with intersecting pathways (72). This connection is crucial in cancer immunotherapy. CD8^+^ T cells can not only induce apoptosis via the granzyme-perforin pathway but also trigger tumor cell ferroptosis by releasing IFN-γ, which downregulates SLC7A11 (73). Furthermore, inflammatory signals from ferroptotic cells can remodel the tumor microenvironment, converting “cold” tumors into “hot” ones, thereby enhancing the efficacy of immune checkpoint inhibitors (74, 75). In glioblastoma, ferroptosis, pyroptosis, and autophagy form a complex regulatory network influencing tumor progression and treatment response (76).

#### 2.6.4 Comparison and interaction with cuproptosis

As two novel metal-dependent regulated cell death pathways, ferroptosis (iron-driven lipid peroxidation) and cuproptosis (excess copper leading to aggregation of lipoylated TCA cycle proteins and proteotoxic stress) have distinct core mechanisms (77, 78). Despite this, significant metabolic interactions exist.

In liver cancer therapy, ferroptosis inducers were found to enhance cuproptosis induced by copper ionophores. The mechanism involves inhibition of mitochondrial protease-mediated degradation of FDX1 (a key cuproptosis protein) and reduced synthesis of glutathione (a major copper chelator) via cystine uptake blockade, collectively promoting copper-dependent lipoylated protein aggregation (79). In cardiovascular diseases, copper can directly promote ferroptosis by inhibiting GPX4. Both ferroptosis and cuproptosis can contribute to PANoptosis—a coordinated cell death involving pyroptosis, apoptosis, and necroptosis—through ROS generation, exacerbating chemotherapy-related cardiotoxicity (80). Epigenetic regulation, such as DNA methylation and non-coding RNAs, has also been identified to modulate key proteins in both ferroptosis and cuproptosis, offering new perspectives for co-targeting (81, 82). Notably, copper metabolism also influences immune cell function, such as T cell activation and macrophage polarization, and may synergize with ferroptosis to enhance immunogenic cell death and reshape the tumor immune microenvironment, thereby providing a rationale for combined immunomodulatory strategies.

## 3 The regulatory network of ferroptosis

Following the exploration of the complex crosstalk between ferroptosis and other cell death modalities, we now delve into its intrinsic regulatory network, emphasizing the pivotal functions of transcription factors and signaling pathways.

### 3.1 Hierarchical regulation by key signaling pathways

Susceptibility to ferroptosis is meticulously governed by several core signaling pathways. The transcription factor NRF2 is a master regulator of the cellular antioxidant response. It confers potent ferroptosis resistance by upregulating key target genes. These include the System Xc light chain subunit *SLC7A11*, the ferritin heavy chain *FTH1*, and a suite of phase II detoxifying enzymes (22, 83).

The tumor suppressor *p53* acts as a context-dependent “double-edged sword” in ferroptosis regulation. It can promote ferroptosis by transcriptionally repressing *SLC7A11*, thereby severing the supply for GSH synthesis. In certain contexts, it may also induce genes like *SAT1* or *GLS2*, indirectly exerting either pro-death or anti-death effects (84, 85).

Furthermore, the Hippo pathway effectors YAP/TAZ are established as significant ferroptosis regulators. Their activation transcriptionally upregulates *ACSL4* and potentially suppresses *FSP1* expression, collectively sensitizing cells to ferroptosis (86, 87). Additional pathways such as AMPK, mTOR, and ATF4 contribute through distinct mechanisms, forming a multi-dimensional regulatory landscape (88–90). These key signaling pathways often have dual roles in regulating both ferroptosis and immune cell function. For example, NRF2 and YAP/TAZ signaling can modulate cytokine production and immune cell polarization, indicating that targeting these pathways may coordinately affect tumor cell death and the immune microenvironment.

#### 3.1.1 The NRF2 pathway

Nuclear factor erythroid 2-related factor 2 (NRF2) is a central regulator of the antioxidant response and plays a core role in inhibiting ferroptosis (91). Under oxidative stress, NRF2 escapes KEAP1-mediated degradation, translocates to the nucleus, and coordinates cellular redox homeostasis, iron metabolism, and lipid peroxidation defense. It does this by activating genes containing antioxidant response elements (AREs), thereby constructing a multi-layered ferroptosis resistance network.

Mechanistically, NRF2 orchestrates key facets of ferroptosis through coordinated regulation of multiple pathways. In glutathione metabolism, it directly upregulates SLC7A11 to enhance cystine uptake for GSH synthesis (92) and activates genes for GSH *de novo* synthesis, supporting GPX4 function (91). Regarding iron metabolism, NRF2 induces expression of FTH1 and heme oxygenase-1, promoting iron storage and detoxification to limit the labile iron pool and reduce Fenton reactions (3, 93). Additionally, NRF2 modulates lipid metabolic enzymes like ACSL4 and SCD1, altering membrane phospholipid composition and reducing susceptibility to lipid peroxidatio (88, 94–96).

Pathophysiologically, aberrant NRF2 pathway activation is closely linked to ferroptosis resistance in cancer cells. In models of ovarian and liver cancer, NRF2 signaling enhances resistance to inducers like erastin and sorafenib (97, 98), whereas its loss or inhibition increases sensitivity. Notably, oncogenic mutations such as KRASG12D and BRAFV619E can directly induce NRF2 expression, building a robust antioxidant defense system, which offers new insights into tumor drug resistance.

**Figure 4:**
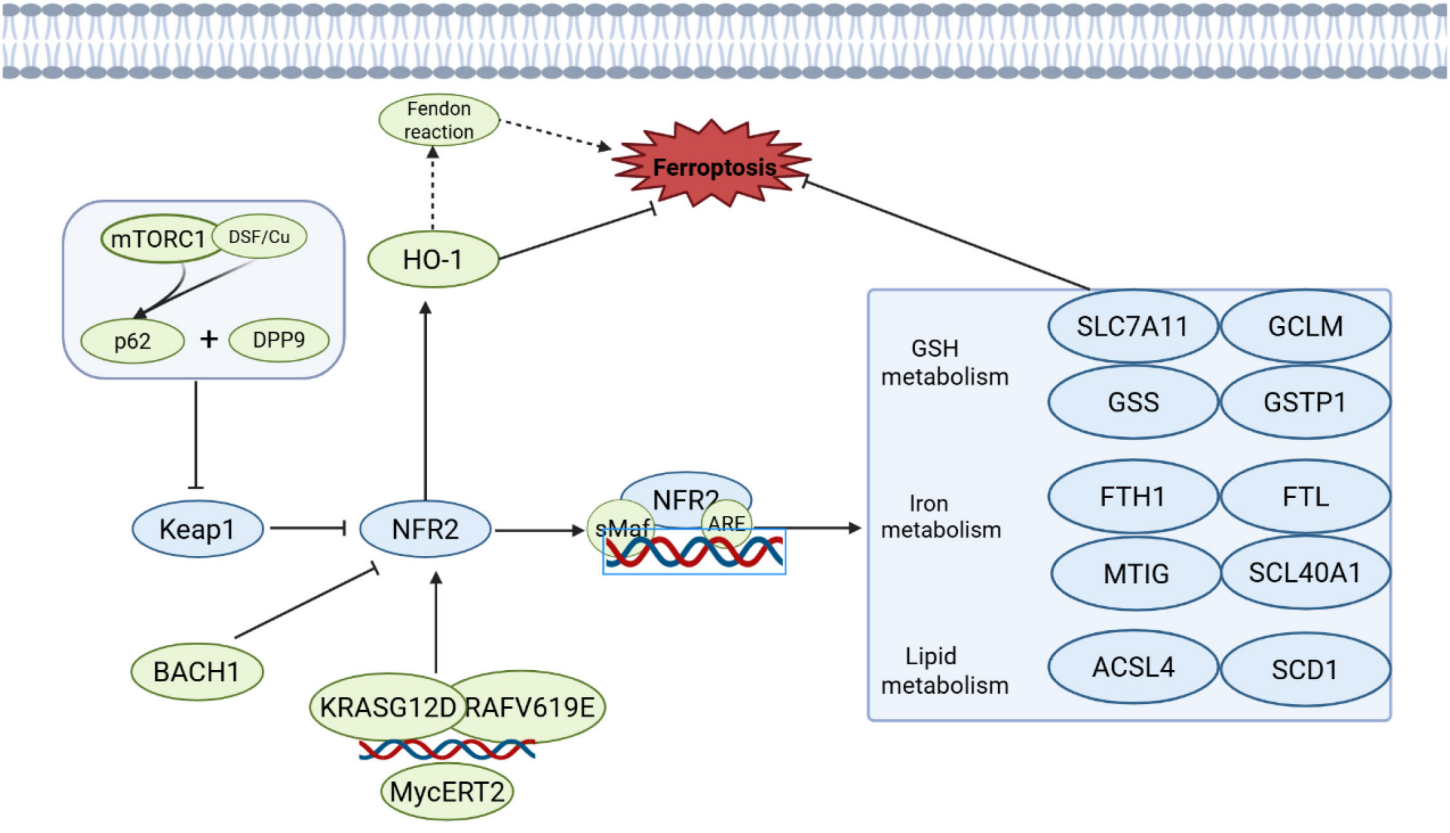
The NRF2-mediated ferroptosis signaling pathway. In the pathway illustrated on the left, mTORC1, p62, and DPP9 interact to modulate NRF2 activity by regulating Keap1. NRF2 functions as a key transcription factor that associates with sMaf and binds to the Antioxidant Response Element (ARE), thereby activating the expression of its downstream target genes. The activity of this pathway is antagonized by BACH1, while it is positively regulated by oncogenic signals such as KRASG12D, RAF V619E, and MycERT2. NRF2 activation induces the expression of HO-1 (heme oxygenase 1), which subsequently influences the Fenton reaction - an iron-dependent process driving lipid peroxidation - thereby contributing to the regulation of ferroptosis.

NRF2 activity itself is subject to precise, multi-layered control. p62 accumulation competitively binds KEAP1, promoting NRF2 stabilization and nuclear translocation. DPP9 cooperates with p62 to enhance NRF2 stability, mediating sorafenib resistance. mTORC1 and disulfiram/copper (DSF/Cu) activate p62 phosphorylation, strengthening KEAP1 inhibition and leading to NRF2 accumulation and ferroptosis resistance (99, 100). Conversely, the transcription factor BACH1 antagonizes NRF2 by suppressing genes like SLC7A11 and FTH1, thereby promoting ferroptosis (87, 101) (**Figure 4**).

Therapeutically, targeting the NRF2 pathway has become a key strategy for reversing tumor ferroptosis resistance. Inhibitors like ML385 significantly enhance the anti-tumor efficacy of ferroptosis inducers combined with chemotherapy, radiotherapy, or immunotherapy (102, 103). However, a major challenge remains achieving tumor-specific targeting due to NRF2’s vital protective role in normal tissues (**Figure 4**).

In summary, NRF2 integrates diverse metabolic and stress signals to establish a sophisticated transcriptional and metabolic defense network. It serves as a critical regulatory hub for ferroptosis susceptibility. A deeper understanding of its regulatory mechanisms and tumor-specific activation will provide a crucial foundation for developing novel ferroptosis-targeted therapies.

#### 3.1.2 The *p53* pathway

As a paramount tumor suppressor, *p53* exhibits a complex and sophisticated “double-edged sword” role in regulating ferroptosis, with its function being highly dependent on cell type, stress signal intensity, and microenvironmental context (85, 104).

The most established pro-ferroptotic function of *p53* involves the transcriptional repression of cystine uptake. *P53* directly binds to the SLC7A11 promoter, repressing its transcription and consequently limiting extracellular cystine import (4). This action impedes intracellular glutathione (GSH) synthesis, thereby weakening GPX4 activity and sensitizing cells to lipid peroxidation accumulation and ferroptosis. Notably, transcription-independent functions of *p53* also contribute to ferroptosis regulation. *p53* can facilitate the nuclear translocation of the deubiquitinase USP7, leading to reduced levels of histone H2B monoubiquitination at lysine 120 (H2Bub1), an epigenetic mark crucial for SLC7A11 transcriptional activation. This decrease in H2Bub1 further reinforces SLC7A11 suppression, deepening the commitment to ferroptosis (105). Beyond SLC7A11, *p53* can indirectly promote lipid peroxidation by activating other downstream targets. For instance, *p53* transcriptionally upregulates spermidine/spermine N1-acetyltransferase 1 (SAT1), driving polyamine metabolism, a process associated with increased arachidonate 15-lipoxygenase (ALOX15) activity, collectively fostering lipid hydroperoxide generation (106). Simultaneously, *p53*-induced expression of glutaminase 2 (GLS2) enhances glutaminolysis, potentially influencing ferroptosis sensitivity by altering the intracellular redox state or providing precursors for lipid synthesis (85, 107). These parallel pro-death pathways collectively enable *p53* to effectively induce ferroptosis, fulfilling its tumor-suppressive function.

**Figure 5:**
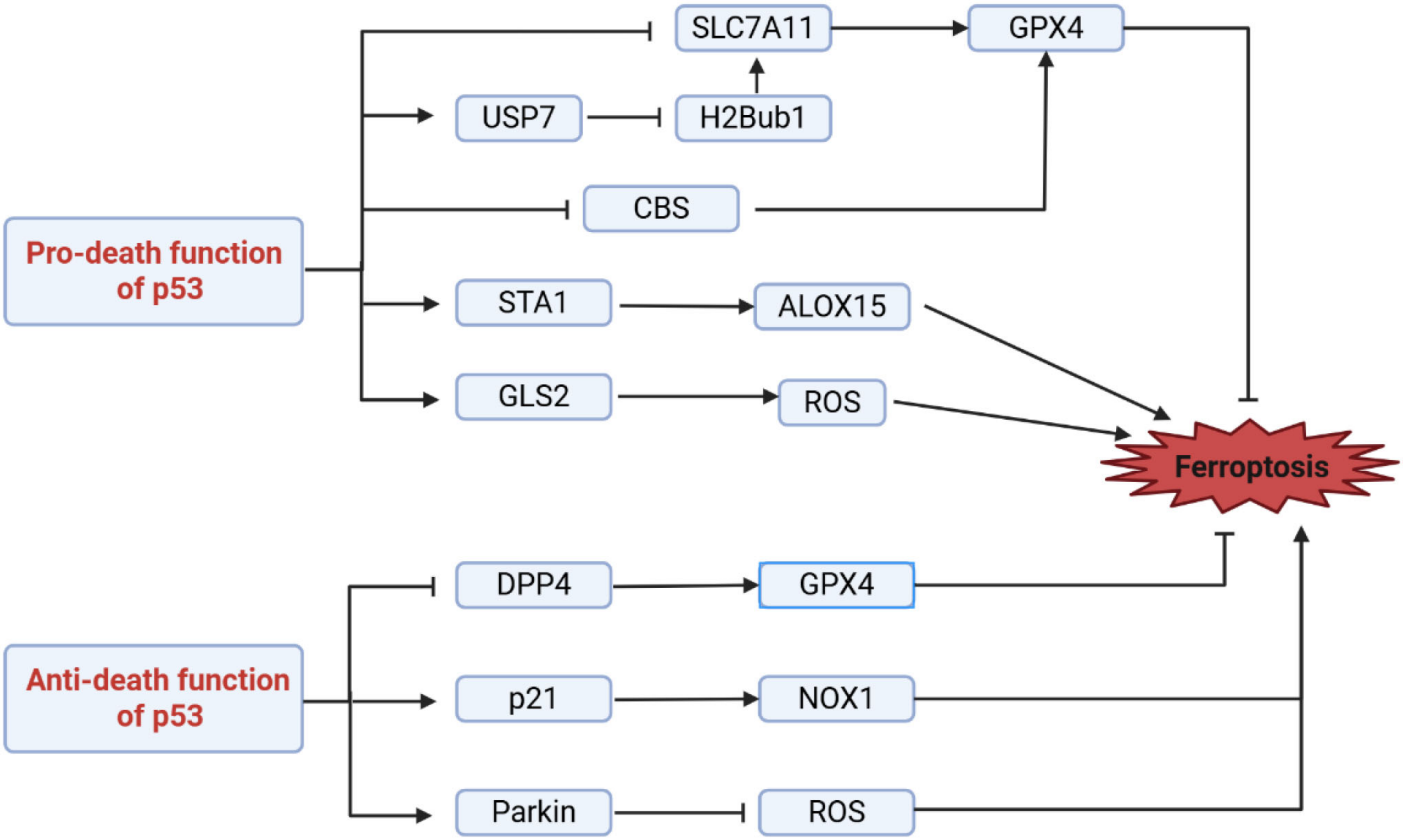
The dual role of p53 in regulating ferroptosis. p53 exerts opposing effects on ferroptosis through distinct molecular mechanisms. On one hand, it can promote ferroptotic cell death by repressing the expression of SLC7A11 and cystathionine beta-synthase (CBS), or alternatively by activating the expression of SAT1 and GLS2. Conversely, p53 can suppress ferroptosis through alternative pathways, either by inhibiting DPP4 activity or via the induction of CDKN1A/p21 and Parkin expression.

Conversely, under specific cellular contexts or stress levels, *p53* demonstrates clear anti-ferroptotic effects. This protective role is partly mediated via transcription-independent mechanisms. Nuclear *p53* can interact with and sequester dipeptidyl peptidase 4 (DPP4), preventing its translocation to the plasma membrane. As membrane-localized DPP4 is required for NADPH oxidase-dependent lipid peroxidation, *p53*-mediated nuclear sequestration effectively inhibits ferroptosis initiation (19). Alternatively, classical transcription-dependent functions also contribute to the protective mechanism. *P53* can induce the expression of cyclin-dependent kinase inhibitor 1A (CDKN1A/p21), leading to cell cycle arrest. This proliferative halt may indirectly reduce basal levels of cellular anabolism and reactive oxygen species (ROS) generation, thereby enhancing resistance to ferroptosis (108). Furthermore, *p53* has been reported to suppress mitophagy. In certain contexts, p53 binds to and sequesters Parkin in the cytoplasm, hindering its translocation to damaged mitochondria and thus inhibiting their clearance. Since damaged mitochondria are a significant source of ROS, preventing their autophagic removal may help maintain mitochondrial fitness and reduce pro-ferroptotic oxidative stress signals (109) (**Figure 5**).

#### 3.1.3 The YAP/TAZ pathway

As core effectors of the Hippo signaling pathway, YAP and TAZ play a critical yet complex role in regulating ferroptosis. Notably, their function exhibits a distinct context-dependent duality, influenced by factors such as cell type, tumor microenvironment, and oncogenic background.

**Figure 6:**
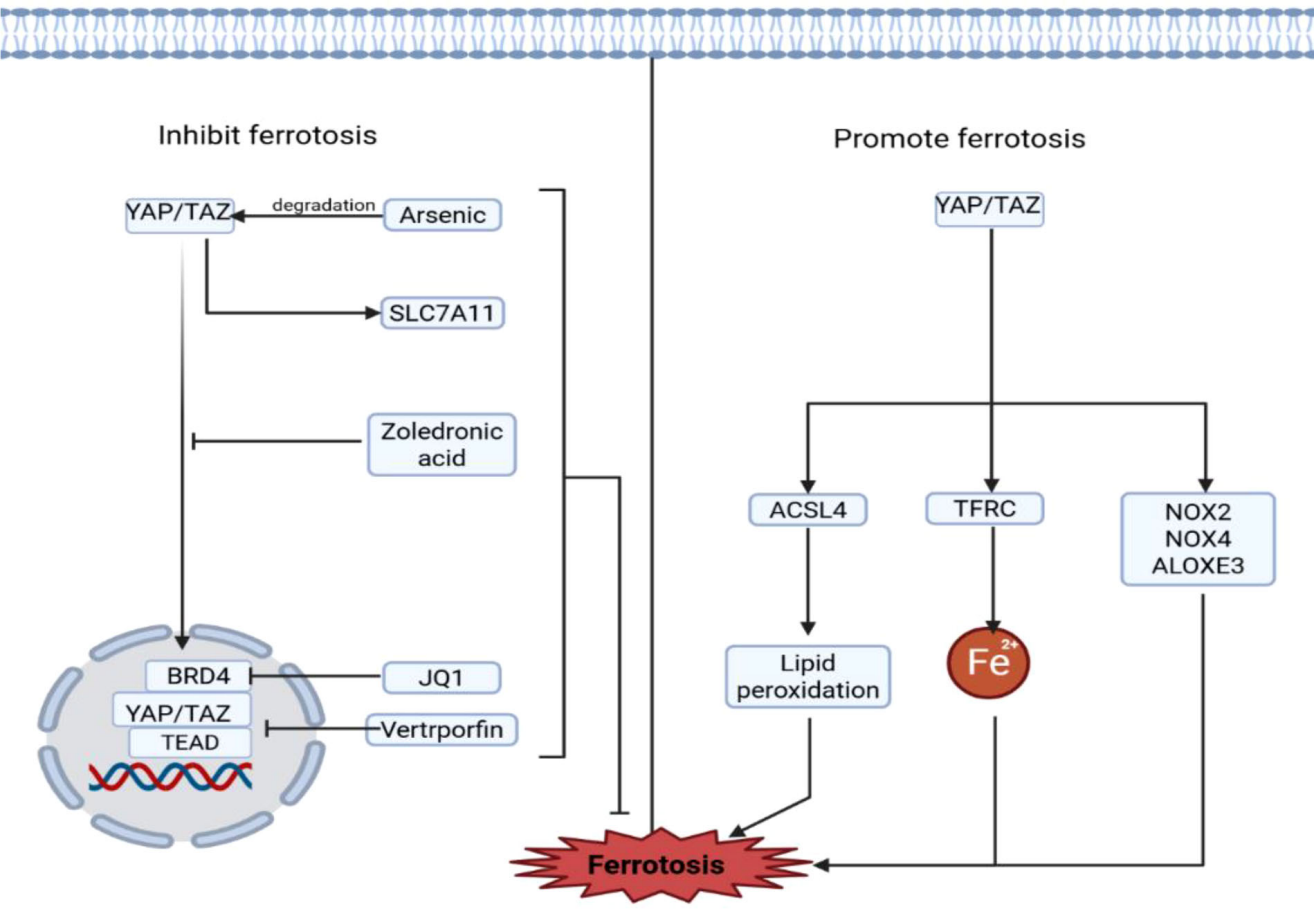
Mechanisms of YAP signaling in regulating ferroptosis.

In most scenarios, YAP/TAZ activation promotes ferroptosis through multiple mechanisms. Specifically, YAP can increase intracellular iron concentration by transcriptionally upregulating transferrin receptor expression (110). Concurrently, YAP activation promotes lipid peroxidation by modulating arachidonate lipoxygenase 3 (111). More significantly, YAP/TAZ enhance cellular sensitivity to ferroptosis by upregulating ACSL4 expression (112). Consistently, studies in renal fibrosis also link YAP activation to elevated ACSL4 levels and increased ferroptosis susceptibility (113). TAZ, on the other hand, indirectly regulates the expression of ROS-producing NADPH oxidases via the angiopoietin-like 4 (ANGPTL4)-NOX2 axis and the epithelial membrane protein 1 (EMP1)-NOX4 axis, further promoting lipid peroxidation and ferroptosis (114). Additionally, the YAP/p53 axis has been demonstrated as essential for cytoglobin-induced lipid peroxidation and ferroptosis (115) (**Figure 6**).

Conversely, in certain specific cancer types, YAP/TAZ exhibit an opposing, ferroptosis-suppressive role. For instance, in sorafenib-resistant hepatocellular carcinoma, YAP/TAZ induce SLC7A11 expression in a TEAD-dependent manner, helping cells resist ferroptosis (86) (**Figure 6**).

For cancers where YAP/TAZ inhibit ferroptosis, various targeted inhibitors have shown promising therapeutic potential. Mevalonate pathway inhibitors, such as zoledronic acid, suppress YAP/TAZ nuclear translocation by maintaining them in a phosphorylated state (116). Verteporfin inhibits the transcriptional activity of YAP/TAZ by disrupting their interaction with TEAD. The BET protein inhibitor JQ-1 suppresses YAP-mediated transcription by targeting BRD4, a key component of the YAP/TAZ-TEAD complex (116). In esophageal squamous cell carcinoma, arsenic-iron oxide conjugate nanocomposites effectively degrade YAP, significantly sensitizing tumor cells to radiotherapy and chemotherapy (117). These inhibitors disrupt the YAP/TAZ signaling pathway through distinct mechanisms, successfully inducing ferroptosis in specific cancer types and offering novel strategies to overcome therapy resistance (**Figure 6**).

YAP signaling can suppress ferroptosis by targeting the ferritin light chain and SLC7A11. Inhibition of YAP/TAZ activity can be achieved through several approaches: mevalonate pathway inhibitors (e.g., zoledronic acid) maintain YAP/TAZ in a phosphorylated state; verteporfin inhibits the interaction between YAP/TAZ and TEAD, thereby preventing DNA binding and transcription; and JQ1 inhibits BRD4, a component of the YAP/TAZ-TEAD complex. Conversely, YAP activation promotes ferroptosis by upregulating the expression of ACSL4 and TFRC. Additional regulators of YAP-mediated ferroptosis include NOX2, NOX4, ALOXE3, and cytoglobin.

### 3.2 Functional network of core regulators

The execution of ferroptosis is governed by a dynamic balance between pro-ferroptotic and anti-ferroptotic factors. Among the pro-ferroptotic regulators, ACSL4 acts as a decisive hub by catalyzing the formation of PUFA-CoAs, thereby supplying the essential substrates for lipid peroxidation. Its absence can completely block erastin-induced ferroptosis (118). Concurrently, NCOA4-mediated ferritinophagy amplifies the Fenton reaction by releasing free iron (119) (**Table 1**).

**Table 1:** Ferroptosis-associated regulatory factors.

Category	Factor	Mechanism of action	Therapeutic significance in oncology
Pro-ferroptotic factor	ACSL4	Catalyzes the synthesis of PUFA-CoA,thereby increasing the susceptibility of membrane phospholipids to peroxidation.	Highly expressed in Epithelial-Mesenchymal Transition (EMT) cells; targeted induction can eliminate metastatic cancer cells (124)
	NCOA4	Mediates ferritinophagy, releasing free iron to fuel the Fenton reaction.	Ataxia Telangiectasia Mutated (ATM) inhibitors suppress ferroptosis by blocking NCOA4 phosphorylation and synergize with ferroptosis inducers to enhance efficacy (119)
	ALOX15/15B	Directly oxidizes PUFA phospholipids, initiating the lipid peroxidation chain reaction.	p53 activates ALOX15 to promote ferroptosis, showing therapeutic relevance in p53 wild-type tumors (83)
	BECN1	Binds to SLC7A11 to inhibit System Xc^−^ activity, thereby reducing GSH synthesis.	Autophagy agonists (e.g., rapamycin) enhance sensitivity to ferroptosis (122)
Anti-ferroptotic factor	GPX4	Reduces lipid hydroperoxides (LOOH) to alcohols, thereby blocking lipid peroxidation.	Inhibitors like RSL3 can overcome chemotherapy resistance (125)
	FSP1	Reduces CoQ10 to CoQH_2_, which traps lipid radicals (independently of GPX4).	FSP1 inhibitor iFSP1 cooperates with GPX4 inhibitors to synergistically kill resistant tumors (27)
	SLC7A11 (xCT)	The light chain subunit of System Xc^−^, mediating cystine uptake for GSH synthesis.	Sorafenib and erastin exert their effects by targeted inhibition, thereby enhancing the therapeutic response in liver cancer (85)
	NRF2	Transcriptionally activates SLC7A11, FTH1, and HERC2/VAMP8 to maintain iron homeostasis.	NRF2 inhibitors enhance sensitivity to ferroptosis and overcome resistance in ovarian cancer (126)
	MBOAT1/2	A phospholipid remodeling enzyme that reduces membrane PUFA content (functions independently of GPX4/FSP1).	Sex hormone receptor antagonists (e.g., tamoxifen) downregulate MBOAT to sensitize breast cancer cells to ferroptosis (29)
	GSTP1	A selenium-independent glutathione peroxidase that detoxifies lipid hydroperoxides (constituting a third defensive axis).	SMURF2 promotes GSTP1 degradation and sensitizes tumors to PD-1 blockade (127)
Dual regulator	p53	Pro-death: Inhibits *SLC7A11* and activates *ALOX15*; Anti-death: Induces expression of *CDKN1A* and *GPX4* (tissue-dependent)	Tumors with mutant p53 require combination therapy with ferroptosis inducers (84)
	STAT3	Pro-death: Downregulates *GPX4*/*SLC7A11* upon its loss/deficiency. Anti-death: Activates transcription of *GPX4*/*SLC7A11*/*FTH1* under resistant conditions.	The STAT3 inhibitor W1131 reverses chemotherapy resistance in gastric cancer (128)

The defensive machinery extends beyond the canonical GPX4 axis. The FSP1-CoQ10-NAD(P)H system functions as a radical-trapping antioxidant, with its expression regulated by transcription factors like NF-κB and miRNAs such as miR-4443 (120). The GCH1-BH4 pathway exerts its protective effect by stabilizing the plasma membrane, while DHODH maintains the mitochondrial CoQH_2_ pool (28). Notably, autophagy and the ubiquitin system exert bidirectional control. Selective autophagy promotes ferroptosis by degrading ferritin or lipid droplets (121, 122), whereas the ESCRT-III complex enhances resistance by repairing plasma membrane damage (90). A recently identified third defense axis involves GSTP1, which detoxifies lipid hydroperoxides via a selenium-independent glutathione peroxidase activity. The degradation of GSTP1, mediated by the E3 ubiquitin ligase SMURF2, constitutes a protective pathway parallel to GPX4 and FSP1 (123). Many of these core regulators are expressed not only in tumor cells but also in immune cells, where they influence cell survival, activation, and functional polarization. A comprehensive understanding of their roles in both compartments is essential for designing therapies that selectively induce ferroptosis in tumors while preserving or enhancing immune function (**Table 1**).

## 4 The role of ferroptosis in tumor biology and immune regulation

### 4.1 The role of ferroptosis in tumor biology

The relationship between ferroptosis and tumor biology is complex and paradoxical. On one hand, it functions as an intrinsic tumor-suppressive mechanism, eliminating early-stage tumor cells or those under specific stresses. On the other hand, during tumor evolution, cancer cells may also exploit or evade ferroptosis to gain a growth advantage (1).

During tumor initiation and development, the view that ferroptosis primarily acts as a tumor suppressor is predominant. The functions of multiple classical tumor suppressor genes have been found to be directly linked to inducing ferroptosis sensitivity. A prime example is p53; beyond its canonical role in regulating apoptosis, activated p53 can increase cellular susceptibility to ferroptosis by transcriptionally repressing the expression of SLC7A11 (4). Similarly, loss of BAP1 upregulates SLC7A11 expression by altering histone modifications, thereby promoting tumorigenesis and enhancing resistance to ferroptosis (129). These studies indicate that evading ferroptosis is a crucial step for certain cancer cells to achieve malignant transformation. However, under specific tumor microenvironment (TME) stresses, such as hypoxia or nutrient deprivation, inducing ferroptosis might also clear a portion of cancer cells, provide nutrients for surviving cells, or affect neighboring cells through a bystander effect, indirectly promoting the adaptive evolution of tumors, although direct evidence for this is still accumulating (130). Therefore, the role of ferroptosis in tumorigenesis is highly context-dependent, but its position as an important natural anticancer barrier is widely recognized (2).

In contrast to its complex role in tumor initiation, the function of ferroptosis in suppressing tumor metastasis is becoming increasingly clear. Metastasis is an inefficient process, with the vast majority of tumor cells entering the circulation undergoing apoptosis or anoikis. Recent studies have found that many circulating tumor cells (CTCs) and disseminated tumor cells are resistant to traditional apoptosis but exhibit high sensitivity to ferroptosis. These cells often undergo an epithelial-mesenchymal transition, characterized by loss of cell-cell junctions and enhanced migratory and invasive capabilities (131). Furthermore, CTCs detached from the extracellular matrix (ECM) face anoikis stress, leading to lipid peroxide accumulation and a lowered threshold for ferroptosis (132). In preclinical models, using GPX4 inhibitors or SLC7A11 inhibitors effectively induced ferroptosis in CTCs and significantly suppressed distant metastasis (133). Consequently, targeting ferroptosis offers an “Achilles’ heel” strategy to specifically eliminate those cancer cell populations with high metastatic potential.

One of the most severe challenges in current cancer therapy is treatment resistance. Numerous studies have shown that cancer cells resistant to chemotherapy, radiotherapy, targeted therapy, and even immunotherapy often exhibit high dependence on ferroptosis. Drug-resistant cancer cells, particularly those persister cells that have acquired a mesenchymal-like or dedifferentiated state, typically upregulate multiple antioxidant defense systems (134, 135).

### 4.2 Ferroptosis and the tumor immune microenvironment

The interaction between ferroptosis and the tumor immune microenvironment (TME) forms a dynamic regulatory network, where immune cells are not only regulators of ferroptosis but their own functions are also profoundly influenced by it. This understanding provides a novel theoretical basis for combination immunotherapy.

#### 4.2.1 Ferroptosis and CD8^+^ T cells

The role of CD8^+^ T cells in tumor immunotherapy extends beyond direct tumor cell killing to include the regulation of a novel cell death modality—ferroptosis. Research shows that CD8^+^ T cells activated by immunotherapy can downregulate the expression of the key cystine transporter SLC7A11 (a component of system Xc^−^) in tumor cells by releasing interferon-gamma (*IFN-γ*). This limits cystine uptake, leading to lipid peroxide accumulation and inducing ferroptosis in tumor cells (136). Radiotherapy and immunotherapy can act synergistically to enhance tumor lipid oxidation and ferroptosis by suppressing SLC7A11, improving tumor control outcomes (137). Furthermore, IFN-γ from CD8^+^ T cells cooperates with fatty acids like arachidonic acid in the TME, driving tumor cell ferroptosis by upregulating the key lipid metabolism enzyme ACSL4 (138).

**Figure 7:**
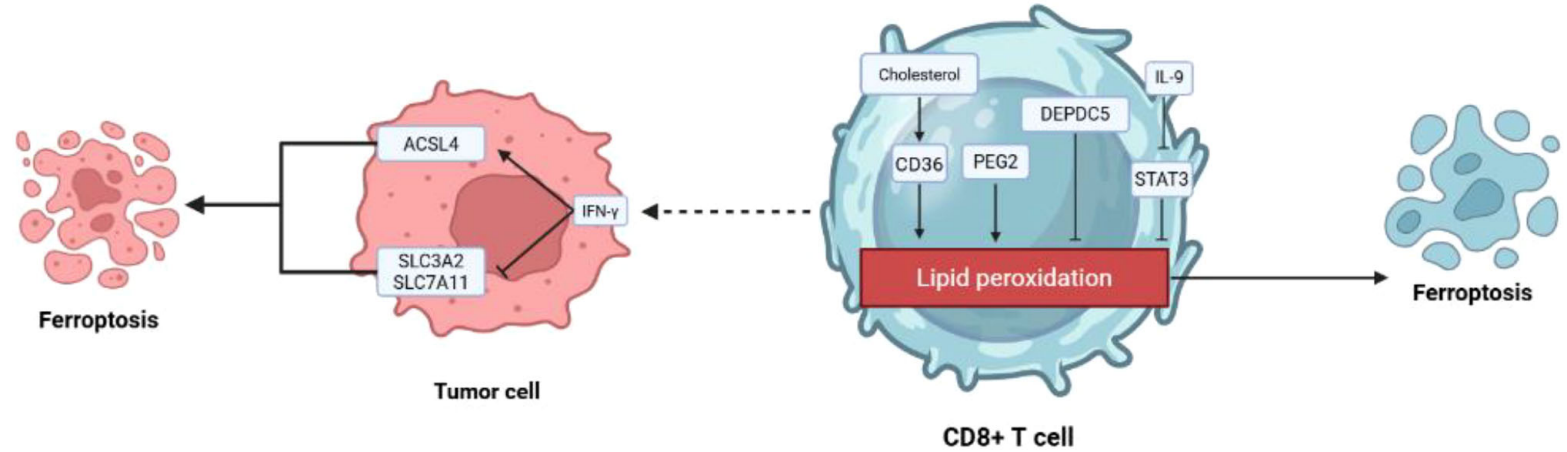
Schematic diagram of the interaction between ferroptosis and CD8^+^ T cells in the TME. This illustration summarizes the dual roles in the interplay between CD8^+^ T cells and tumor cells via ferroptosis: ① The Effector Role: CD8^+^ T cells induce ferroptosis in tumor cells by downregulating SLC7A11 and upregulating ACSL4 via IFN-γ; ②The Target Role: CD8^+^ T cells themselves are susceptible to ferroptosis within the tumor microenvironment due to lipid metabolism dysregulation (e.g., CD36, PGE2) and signaling abnormalities (e.g., DEPDC5 deficiency), leading to functional exhaustion.

Conversely, the TME can actively induce ferroptosis in CD8^+^ T cells, impairing their anti-tumor function. For instance, cholesterol in tumors can cause CD8^+^ T cell exhaustion by triggering endoplasmic reticulum stress and activating XBP1 (139). More directly, the fatty acid transporter CD36 on the surface of CD8^+^ T cells mediates excessive uptake of fatty acids from the TME, directly triggering intracellular lipid peroxidation and ferroptosis, compromising their cytokine production capability and anti-tumor function (140). Similarly, prostaglandin E2 (PGE2) can promote ferroptosis in tumor-infiltrating CD8^+^ T cells by interfering with the IL-2 signaling pathway (141). Within T cells, loss of the DEPDC5 gene leads to overactivation of mTORC1 signaling, upregulating the expression of ATF4 and xanthine oxidase, thereby spontaneously inducing ferroptosis in CD8^+^ T cells (142) (**Figure 7**).

Based on these mechanisms, enhancing the persistence of CD8^+^ T cells and protecting them from ferroptosis has emerged as a strategy to improve immunotherapy efficacy. Studies have found that the Tc9 subset (IL-9-secreting CD8^+^ T cells) reduces its own lipid peroxidation and ferroptosis by enhancing fatty acid oxidation via the IL-9/STAT3 signaling axis, allowing it to survive longer in the TME and exhibit stronger anti-tumor capabilities (143). Therapeutically, targeting HnRNP L can downregulate PD-L1 expression on tumor cells while promoting CD8^+^ T cell-mediated tumor ferroptosis, synergizing with anti-PD-1 therapy (144). In liver cancer, restoring MAT1A expression has also been shown to enhance CD8^+^ T cell activity and induce tumor cell ferroptosis (145). Collectively, these findings highlight the central role of ferroptosis in T cell immunity, providing a theoretical foundation for combining immune checkpoint blockade with ferroptosis-inducing strategies.

#### 4.2.2 Ferroptosis and macrophages

The occurrence of ferroptosis itself constitutes a potent cellular stress signal, actively reprogramming the functional phenotype of macrophages towards a pro-inflammatory, anti-tumor M1 direction. The molecular basis of this process lies in the core biochemical reaction of ferroptosis—lipid peroxidation. Studies confirm that in nasopharyngeal carcinoma models, the enzyme ACSL4 promotes ferroptosis by driving lipid peroxidation, which directly leads to the repolarization of tumor-associated macrophages (*TAM*) from a pro-tumor M2 phenotype to an anti-tumor M1 phenotype (146). Similarly, in the acidic microenvironment of breast cancer, ferroptosis activated by the ZFAND5/SLC3A2 signaling axis not only directly kills tumor cells but also acts as a polarization signal, inducing macrophage transformation into the M1 phenotype, forming a “dual-strike” mechanism against the tumor (147). More interestingly, the susceptibility of macrophages themselves to ferroptosis determines their functional state. For instance, classically activated M1 macrophages generate large amounts of nitric oxide (NO•) through high expression of inducible nitric oxide synthase(*iNOS*). NO• can competitively inhibit the activity of the 15-lipoxygenase/PEBP1 complex, thereby blocking the generation of the key pro-ferroptotic phospholipid HpETE-PE. This intrinsic anti-ferroptosis mechanism allows M1 macrophages to survive in the pro-inflammatory environment and continuously perform immune surveillance functions, constituting their unique cellular identity (148, 149). Therefore, the ferroptosis pathway is both a result and a significant driver of macrophage polarization.

In the tumor microenvironment, through direct and indirect intercellular communication, macrophages become key arbiters determining whether tumor cells undergo ferroptosis, with a distinctly bidirectional regulatory role. On one hand, macrophages can act as “promoters” of ferroptosis. In liver and lung cancer models, the absence of the cystine transporter xCT in macrophages triggers their own ferroptosis and inhibits M2 polarization. This change remodels the immune microenvironment, recruiting and activating more CD8^+^ T cells. The activated T cells, by secreting IFN-γ, subsequently downregulate System Xc^−^ activity on tumor cells, making them more sensitive to ferroptosis, thereby amplifying the cascade of anti-tumor immunity (150, 151). On the other hand, macrophages may also transform into “resistors” of ferroptosis, leading to therapeutic failure. Recent studies have found that when ferroptosis inducers are used, macrophages themselves undergo phospholipid peroxidation. This lipid peroxidation impairs their phagocytic function mediated by TLR2, preventing them from effectively clearing tumor cells that have undergone ferroptosis, ultimately resulting in tumor resistance to ferroptosis therapy (152). Furthermore, in some cases, low-dose ferroptosis inducers (such as erastin) fail to kill tumor cells. Instead, by activating the STAT3/IL-8 signaling axis in macrophages, they polarize macrophages into an M2 phenotype, thereby enhancing tumor invasion and metastasis, completely contrary to the therapeutic intent (153). This demonstrates that the functional state of macrophages is a core variable determining the success or failure of ferroptosis therapy.

#### 4.2.3 Ferroptosis and myeloid-derived suppressor cells

Myeloid-derived suppressor cells (*MDSCs*) maintain their immunosuppressive function in the tumor microenvironment through various molecular mechanisms and exhibit significant resistance to ferroptosis. Mechanistically, the high expression of N-acylsphingosine amidohydrolase 2 (*ASAH2*) in *MDSCs* effectively reduces ROS generation by inhibiting the p53-Hmox1 signaling axis, thereby protecting cells from ferroptosis (154). Concurrently, *MDSCs* undergo unique lipid metabolic reprogramming. The uptake of arachidonic acid mediated by fatty acid transport protein 2 (*FATP2*) and the synthesis of prostaglandin E2 significantly reduce lipid peroxide accumulation, further enhancing resistance to ferroptosis (155). Additionally, *MDSCs* utilize their highly active Xc^−^ system to take up large amounts of extracellular cystine. While maintaining their own redox homeostasis, this leads to cystine/cysteine depletion in the tumor microenvironment, consequently impairing T cell activation and function (156). These findings reveal that *MDSCs* construct a comprehensive ferroptosis defense system through multiple pathways, including *ASAH2*, lipid metabolic reprogramming, and cystine deprivation, thereby maintaining an immunosuppressive state in the tumor microenvironment. Therefore, targeting these key pathways to induce ferroptosis in *MDSCs* may become an effective strategy for reversing immunosuppression and enhancing anti-tumor immune responses.

Based on the above mechanisms, the synergy between ferroptosis and immune checkpoint inhibitors constitutes a powerful positive feedback loop. Ferroptotic tumor cells act as an “*in situ* vaccine,” efficiently priming dendritic cell-mediated T cell responses by releasing tumor antigens and damage-associated molecular patterns (*DAMPs*) (157, 158). Meanwhile, by eliminating *MDSCs* and promoting M1 macrophage polarization, ferroptosis inducers can effectively reverse “cold tumors” into “hot tumors” infiltrated by immune cells, fundamentally improving the microenvironment for immunotherapy (159). Therefore, targeting ferroptosis defense nodes highly expressed in tumors, such as *SLC7A11*, has become an effective strategy to overcome primary and secondary resistance to immune checkpoint inhibitors (160). Emerging nanotherapeutic strategies (e.g., metal-organic framework nanoparticles loaded with sorafenib) not only induce tumor ferroptosis but also actively promote CD8^+^ T cell activation and tumor infiltration, achieving synergistic enhancement between ferroptosis induction and immune activation (161). Collectively, ferroptosis serves as a potent modulator of the tumor immune landscape. By inducing immunogenic cell death, reprogramming macrophage polarization, and depleting immunosuppressive cells, it can effectively convert immunologically “cold” tumors into “hot,” T-cell-inflamed microenvironments. This remodeling not only enhances intrinsic anti-tumor immunity but also establishes a strong rationale for combining ferroptosis inducers with immune checkpoint inhibitors, offering a promising strategy to overcome resistance and improve therapeutic outcomes.

#### 4.2.4 Integrative perspective: ferroptosis as a multifunctional immune modulator

Ferroptosis functions as a multifunctional immune modulator within the TME, with its net impact on antitumor immunity determined by a balance of cell type-specific effects. This duality is exemplified by CD8^+^ T cells, which can induce tumor ferroptosis (e.g., via IFN-γ) yet are themselves vulnerable to ferroptosis, compromising their effector function. Macrophages primarily act as responders; ferroptotic signals can promote immunostimulatory M1 polarization, but their functional state also dictates therapeutic outcome by influencing immunogenic clearance or pro-tumor M2 skewing. In contrast, myeloid-derived suppressor cells (MDSCs) resist ferroptosis through robust antioxidant defenses, thereby sustaining an immunosuppressive microenvironment.

This analysis highlights that the pro-immunogenic effects of ferroptosis (e.g., DAMP release, M1 polarization) largely stem from targeting tumor cells and certain innate immune cells. Conversely, its immunosuppressive effects often arise from collateral damage to CD8^+^ T cells or the fortification of immunosuppressive networks (MDSCs, M2 macrophages). Therefore, promising therapeutic strategies should aim to selectively induce ferroptosis in tumor and immunosuppressive cells while protecting or reinvigorating CD8^+^ T cells. Future combination therapies must be designed with this cellular selectivity in mind to harness the immunostimulatory potential of ferroptosis while mitigating its detrimental effects on adaptive immunity.

## 5 Novel tumor therapeutic strategies targeting ferroptosis

### 5.1 Direct induction of ferroptosis

#### 5.1.1 Small molecule inducers

Small molecule inducers targeting the core ferroptosis machinery represent the most direct and extensive strategy for activating this cell death pathway. Based on their targets, they are primarily categorized into System Xc^−^ inhibitors, GPX4 inhibitors, and compounds targeting iron metabolism (2).

System Xc^−^ inhibitors work by blocking cystine uptake, depleting intracellular glutathione, and consequently removing support for GPX4 activity. First-generation inhibitors like Erastin and its derivatives, as well as the clinically used drug sulfasalazine, function by inhibiting SLC7A11 activity (1, 133). Recent studies have identified various other compounds and natural products that can indirectly induce ferroptosis by targeting the System Xc^−^ axis.

GPX4 inhibitors directly act on the key enzyme GPX4, inactivating it. RSL3 is the prototype of this class, which irreversibly inhibits GPX4 enzyme activity by covalently binding to its active site selenocysteine (23). Beyond direct inhibitors, regulating GPX4 stability is also an effective approach (162, 163).

Compounds targeting iron metabolism modulate intracellular iron homeostasis, increasing the labile iron pool to catalyze the Fenton reaction and accelerate lipid peroxidation. This includes strategies that utilize iron chelators—which in specific contexts can inhibit ferroptosis by sequestering excess iron—but more commonly involves using iron ionophores or inducing ferritinophagy to increase intracellular free iron.

#### 5.1.2 Nanodelivery systems

Despite the significant potential of small molecule inducers, their clinical application is often limited by poor pharmacokinetic properties, off-target toxicity due to non-specific distribution, and the complexity of the tumor microenvironment (164). Nanotechnology provides revolutionary tools to address these challenges. Engineered nanodelivery systems can significantly enhance the targeting of ferroptosis inducers, reduce side effects, and enable synergy with multiple treatment modalities (165).

Nanocarriers are widely used to efficiently encapsulate and deliver classic small molecule ferroptosis inducers, improving their water solubility, stability, and tumor accumulation. For instance, zwitterionic polymer-coated magnetic nanoparticles loaded with simvastatin were developed for treating triple-negative breast cancer (166).

Inorganic nanomaterials themselves, particularly iron-based nanomaterials, can serve dual roles as both iron sources and Fenton reaction catalysts, inherently inducing ferroptosis. They can dissociate within tumor cells to release Fe²^+^/Fe³^+^, catalyzing H_2_O_2_ to produce highly toxic ·OH and trigger lipid peroxidation (165, 167).

The most advanced strategies involve designing multifunctional nano-platforms that combine ferroptosis induction with other therapeutic modalities for a powerful synergistic antitumor effect. For example, Li et al. designed a core-shell nanoparticle for co-delivering copper and Erastin, successfully synergizing cuproptosis and ferroptosis to enhance cancer immunotherapy (168). Although nanocarriers hold great promise for improving targeting and reducing toxicity, their *in vivo* stability, potential immunogenicity, and large-scale standardized manufacturing remain critical bottlenecks for clinical translation.

### 5.2 Combination therapy strategies

#### 5.2.1 Combination with conventional therapies

Radiotherapy and chemotherapy are mainstays of current cancer treatment, but their efficacy is often limited by acquired resistance in tumor cells. Recent studies indicate that ferroptosis plays a key role in cell death induced by these therapies, particularly in drug-resistant tumor cells with mesenchymal features or enhanced antioxidant capacity.

Radiotherapy generates reactive oxygen species via ionizing radiation, initiating lipid peroxidation and subsequently inducing ferroptosis. For instance, in nasopharyngeal carcinoma, radiotherapy can upregulate GSTM3 expression, which stabilizes USP14 and inhibits GPX4, thereby enhancing radiotherapy-induced ferroptosis (169). Chemotherapeutic agents like cisplatin and oxaliplatin can also indirectly induce ferroptosis by depleting glutathione or inhibiting System Xc^−^ function.

#### 5.2.2 Combination with targeted therapy

Targeted therapies function by specifically inhibiting oncogenic signaling pathways in tumor cells, but single-agent application often fails due to pathway redundancy or feedback activation. Combining ferroptosis inducers with targeted drugs can enhance antitumor effects by synergistically regulating metabolic and oxidative stress states.

For example, in melanoma, combining an AXL inhibitor with a BRAF inhibitor significantly induces ferroptosis, suppresses protective autophagy, enhances apoptosis, and overcomes BRAF inhibitor resistance (170). In hepatocellular carcinoma, while Sorafenib can inhibit System Xc^−^ to induce ferroptosis, it simultaneously activates the Nrf2 pathway leading to resistance; Nrf2 inhibitors can block this feedback mechanism, restoring Sorafenib’s ability to induce ferroptosis (171).

#### 5.2.3 Combination with immunotherapy

Combining ferroptosis induction with immunotherapy is one of the most promising directions in current cancer therapy. Ferroptosis, as a form of immunogenic cell death, can release tumor-associated antigens and damage-associated molecular patterns, promoting dendritic cell maturation and antigen presentation, and activating tumor-specific immune responses by cytotoxic T lymphocytes (158, 172).

This immune activation provides a novel approach to overcome primary or acquired resistance to *ICIs*. Research shows that interferon-gamma is a key molecular bridge connecting *ICIs* to ferroptosis; activated CD8^+^ T cells secrete IFN-γ, which downregulates *SLC7A11* expression in tumor cells, thereby inhibiting cystine uptake and enhancing lipid peroxidation and ferroptosis sensitivity (138, 173).

#### 5.2.4 Combination with other physical therapies

The combination of ferroptosis with physical therapies, particularly photodynamic therapy(*PDT*) and photothermal therapy (*PTT*), demonstrates powerful synergistic antitumor potential. *PDT* uses photosensitizers activated by specific light wavelengths to generate *ROS*, directly killing tumor cells and inducing immunogenic cell death; the produced *ROS* can also serve as substrates for the Fenton reaction, promoting lipid peroxidation and thereby sensitizing and enhancing ferroptosis (174, 175).

The integration of nanotechnology has significantly advanced the translational application of this combination strategy. Multifunctional nano-platforms can co-deliver ferroptosis inducers and photosensitizers/photothermal agents, enabling precise, tumor microenvironment-responsive release (167, 176). For instance, nanoparticles loaded with Fe³^+^ and the photothermal agent polydopamine, upon laser irradiation, not only generate thermal effects but also produce ·OH via the Fenton reaction, achieving simultaneous PTT and ferroptosis therapy (177, 178).

## 6 Clinical translation and challenges

### 6.1 Clinical translation research

We systematically searched two major clinical trial registries, ClinicalTrials.gov and the Chinese Clinical Trial Registry, on October 27, 2025, using the keywords “ferroptosis,” “GPX4,” “SLC7A11,” “system Xc−,” and “FSP1.” As of our search date, a total of two registered clinical studies related to ferroptosis were identified (registration numbers NCT06554392 and NCT06048369). Notably, these trials focus on the identification of GPX4 serum levels (NCT06554392) and carbon nanoparticle-loaded iron therapy for advanced solid tumors (NCT06048369), respectively. It is particularly noteworthy that no clinical studies involving specific inhibitors directly targeting GPX4 or FSP1 have been identified to date, underscoring the early stage of clinical translation for ferroptosis-targeting therapies.

### 6.2 Challenges and future perspectives

Although ferroptosis-targeting antitumor strategies show significant potential, their clinical translation faces several critical challenges. The primary challenge lies in improving selectivity/specificity—determining how to precisely induce ferroptosis in tumor cells without harming normal tissues. Since the core drivers of ferroptosis are also vital in normal physiological processes, non-selective induction may lead to toxicity in crucial organs such as the liver, kidneys, and heart (179, 180).

The lack of biomarkers constitutes another central bottleneck. There is currently a shortage of reliable, clinically applicable biomarkers to predict patient sensitivity to ferroptosis induction, monitor treatment response in real-time, or assess the risk of resistance. Although molecules like transferrin receptor 1 and lipid peroxidation products have been explored as potential ferroptosis markers in research (181, 182), their specificity, sensitivity, and validation within the complex human environment require further in-depth study (183). In particular, immune-relevant biomarkers such as soluble TFR1 (sTFR1) in serum, exosomal GPX4, or circulating lipid peroxidation adducts could reflect both ferroptotic activity and associated immune remodeling, warranting clinical exploration. A clinically usable, ferroptosis-specific biomarker system has yet to be established. Future efforts should integrate lipidomics, radiomics, and liquid biopsies to develop dynamic, quantifiable, and predictive biomarker panels.

Problems with *in vivo* efficacy and pharmacokinetics are equally prominent. Many small-molecule compounds with potent ferroptosis-inducing activity suffer from poor *in vivo* stability, rapid metabolism, low tumor tissue accumulation efficiency, and systemic toxicity, which limit their clinical application (125, 184). Additionally, tumor heterogeneity and adaptive resistance cannot be ignored. Tumor cells can rapidly develop acquired resistance by activating alternative anti-ferroptosis pathways or altering lipid metabolism (128, 179).

To address these challenges, future research must focus on multi-dimensional innovation to achieve clinical breakthroughs in ferroptosis therapy. Developing superior inducers is a fundamental direction, involving the design of novel small molecules and biomacromolecular drugs with higher selectivity, lower off-target toxicity, and improved pharmacokinetic properties (179, 180). Constructing intelligent delivery systems is a key strategy for resolving targeting and toxicity issues.

The identification of precise biomarkers is core to guiding individualized therapy. Integrating multi-omics technologies and advanced molecular imaging to deeply mine reliable markers that can predict ferroptosis sensitivity and reflect treatment response in real-time is an essential path for patient stratification, treatment optimization, and dynamic efficacy monitoring (181, 183). In-depth mechanistic research remains the source of continuous innovation. Further elucidation is needed regarding the interaction network between ferroptosis and other cell death modalities, the role of ferroptosis in tumor stem cell maintenance and eradication, and the precise regulatory mechanisms by which ferroptosis induction affects the functions of various immune cell subsets within the tumor microenvironment (185).

## 7 Conclusion

Ferroptosis, a novel form of programmed cell death driven by iron-dependent lipid peroxidation, has seen its molecular machinery widely elucidated. This spans from the collapse of the core System Xc^−^–GSH–GPX4 defensive axis to the discovery of alternative pathways like FSP1–CoQ10 and GCH1–BH4, and the intricate regulation of iron and lipid metabolism (15, 29, 71). Crucially, research has uncovered its complex interplay with key signaling pathways such as NRF2, p53, and Hippo–YAP, forming a multi-layered regulatory network that determines cellular fate (83, 84, 86).

Within tumor biology, ferroptosis plays a dual role: it functions both as an intrinsic tumor-suppressive mechanism and as a “therapeutic blade” that can be precisely wielded. Of paramount importance is the finding that tumor cells resistant to conventional therapies, or those in an epithelial-mesenchymal transition state with high metastatic potential, often unexpectedly exhibit an “acquired vulnerability” to ferroptosis (131, 133, 134). This provides a novel therapeutic window for reversing drug resistance and suppressing metastasis.

Consequently, ferroptosis-targeting antitumor strategies are demonstrating vibrant potential. From small-molecule inducers directly targeting core pathways to smart nano-platforms enabling precise delivery and combination therapy, ferroptosis-based interventions are advancing from concept toward practice (125, 164). Among these, the most groundbreaking direction involves its strategic combination with immunotherapy. Ferroptosis not only directly kills tumor cells but also, by inducing immunogenic cell death, releases tumor antigens and damage-associated molecular patterns, effectively activating dendritic cells and promoting antitumor immunity by cytotoxic T cells (138, 158).

Despite this promising outlook, the clinical translation of ferroptosis therapies faces several hurdles. Tumor heterogeneity and plasticity can lead to the activation of backup anti-ferroptosis pathways, resulting in acquired resistance (30, 121). The lack of reliable predictive biomarkers, coupled with insufficient *in vivo* delivery efficiency and targeting specificity, also hinders its precise clinical application (182, 183).

Looking forward, ferroptosis research is entering a new phase focused on increased precision and integration, driven by a deeper understanding of its immunomodulatory functions. The next generation of studies should be dedicated to: discovering and validating biomarkers for patient stratification; developing smart nanodelivery systems responsive to the tumor microenvironment to enable tumor-specific ferroptosis induction; and designing novel combination strategies based on a deep understanding of tumor-specific metabolic vulnerabilities (186–188). Key breakthroughs should focus on developing tumor-specific inducers, constructing intelligent responsive nano-delivery systems, establishing clinically translatable biomarker systems, and deeply deciphering the role of ferroptosis in tumor stem cells, the immune microenvironment, and heterogeneous drug resistance. Critically, this research must underscore that ferroptosis is not merely a cell death pathway but a potent immunomodulator. Targeting ferroptosis thus represents a paradigm shift, offering a strategic avenue to reshape the next generation of cancer immunotherapies by converting immunologically “cold” tumors into “hot,” T-cell-inflamed microenvironments and synergizing with existing immunomodulatory agents.
